# Association between DIAPH1 variant and posterior circulation involvement with Moyamoya disease

**DOI:** 10.1038/s41598-023-37665-1

**Published:** 2023-07-03

**Authors:** Shihao He, Xiaokuan Hao, Ziqi Liu, Yanru Wang, Junze Zhang, Xilong Wang, Fei Di, Rong Wang, Yuanli Zhao

**Affiliations:** 1 Department of Neurosurgery, Beijing Tiantan Hospital, Capital Medical University, Beijing, 100070 China; 2 Department of Neurosurgery, Stanford University School of Medicine, 300 Pasteur Drive (R281), Stanford, CA 94305-5327 USA; 3 China National Clinical Research Center for Neurological Diseases, Beijing, 100070 China; 4 Center of Stroke, Beijing Institute for Brain Disorders, Beijing, 100069 China; 5 Beijing Institute of Brain Disorders, Collaborative Innovation Center for Brain Disorders, Capital Medical University, Beijing, 100069 China; 6 Department of Neurosurgery, The Affiliated Children’s Hospital, Capital Institute of Pediatrics, Beijing, 100020 China

**Keywords:** Genetic association study, Neurovascular disorders

## Abstract

Moyamoya disease (MMD) is a chronic and progressive cerebrovascular stenosis or occlusive disease that occurs near Willis blood vessels. The aim of this study was to investigate the mutation of DIAPH1 in Asian population, and to compare the angiographic features of MMD patients with and without the mutation of the DIAPH1 gene. Blood samples of 50 patients with MMD were collected, and DIAPH1 gene mutation was detected. The angiographic involvement of the posterior cerebral artery was compared between the mutant group and the non-mutant group. The independent risk factors of posterior cerebral artery involvement were determined by multivariate logistic regression analysis. DIAPH1 gene mutation was detected in 9 (18%) of 50 patients, including 7 synonymous mutations and 2 missense mutations. However, the incidence of posterior cerebral artery involvement in mutation positive group was very higher than that in mutation negative group (77.8% versus 12%; p = 0.001). There is an association between DIAPH1 mutation and PCA involvement (odds ratio 29.483, 95% confidence interval 3.920–221.736; p = 0.001). DIAPH1 gene mutation is not a major genetic risk gene for Asian patients with moyamoya disease but may play an important role in the involvement of posterior cerebral artery.

## Introduction

Moyamoya disease (MMD) is a rare cerebrovascular disease of unknown etiology, characterized by stenosis or occlusion of the end of the internal carotid artery and the formation of an abnormally thin network of blood vessels near the occlusion artery^1^. MMD exhibits primary clinical symptoms of ischemic and/or hemorrhagic stroke and cognitive impairment, affecting both children and adults, thereby increasing the likelihood of disability and death due to the unavailability of effective treatments addressing its pathogenesis^2,3^.

In recent times, scholars have conducted extensive research on the etiology and pathogenesis of MMD, unearthing numerous promising genes. However, despite these advancements, the precise etiology of moyamoya disease remains indeterminate, underscoring the necessity for in-depth investigation of MMD genes^4^. The emergence of high-throughput sequencing technology identified potential genes associated with moyamoya disease susceptibility, notably RNF213, ACTA2 and MYH11^5–7^. Unfortunately, none of this has been successfully tested in mouse models. Mammalian homolog of Drosophila diaphanous 1 (DIAPH1) is a formin protein encoding the RhoA GTPase mDia, which plays an important role in vascular remodeling and thrombosis. A study showed that the expression of DIAPH1 gene was significantly down-regulated in patients with ischemic stroke^8^, furthermore, a recent genetic association study using whole-gene exon sequencing identified rare damaging variants of DIAPH1 in 24-proband discovery cohort and an 84-proband validation cohort of non–East Asian patients with MMD. These mutations may related to thrombocytopenia and clinical symptoms such as ischemic stroke and angiographical stage^9^. But at present, there is no study on DIAPH1 gene mutation in Asian population, so we report DIAPH1 gene mutation in the Asian patient and further investigate its associated clinical features, in particular the angiography results of the posterior cerebral artery (PCA). Our study will help clarify the genetic background of moyamoya disease in different ethnic groups and provide opportunities for early diagnosis and surgical treatment of moyamoya disease.

## Results

A total of 50 patients were included in our study (Table 1). There were 25 females and 25 males. The median age at diagnosis was 24 years. Of the 50 patients, 4 (8%) had a history of hypertension, 3 (6%) had a history of smoking, almost all participants were found not to have any underlying diseases that could affect the results of the study. Infarction was the most common initial symptom (25, 50%). The others were hemorrhage (17, 34%), TIA (8, 16%). PCA involvement was observed in 12 (23.8%) patients (Table 1).

**Table 1:** Sample demographics of patients with MMD.

Characteristics	Total, n (%)
Number of patients	50
Sex (M : F) ratio, n	25/25
Age, years, median (IQR)	24 (19–35)
History of risk factors, n (%)	
Hypertension	4 (8%)
Diabetes	0
Coronary heart disease	0
Hyperlipidemia	0
Smoking history	3 (6%)
Alcohol taking	0
Clinical manifestation, n (%)	
TIA	8 (16%)
Infarction	25 (50%)
Hemorrhage	17 (34%)
PCA involvement (+), n (%)	12 (23.8%)
Leptomeningeal (−)^a^ (posterior to anterior), n (%)	12 (23.8%)
Unilateral MMD, n (%)	2 (4%)

IQR, interquartile range; TIA, transient ischemia attack; PCA, posterior cerebral artery.

^a^Without compensation of leptomeningeal from posterior to anterior.

### DNA sequencing results

Nine gene mutations were detected in 50 moyamoya disease patients, including 7 synonymous mutations and 2 missense mutations. Of the 7 synonymous mutations, 5 are rs2302102 (p.Gly1193=, c.3579C>T in exon27), one is rs199683670(p.Asp210=, c.630T>C in exon 7), another one is no datebase rs number (p.D349=, c.1047C>T in exon 11). Of the 2 missense mutations, one is rs142480526 (p.A67V, c.200C>T in exon3), another one is no datebase rs number (p.E1120K, c.3358G>A on exon 25). The same mutations in non-east Asians as previously described were not reported, more details are in supplementary materials 1.

### Clinical and DSA features between different group

**Figure 1:**
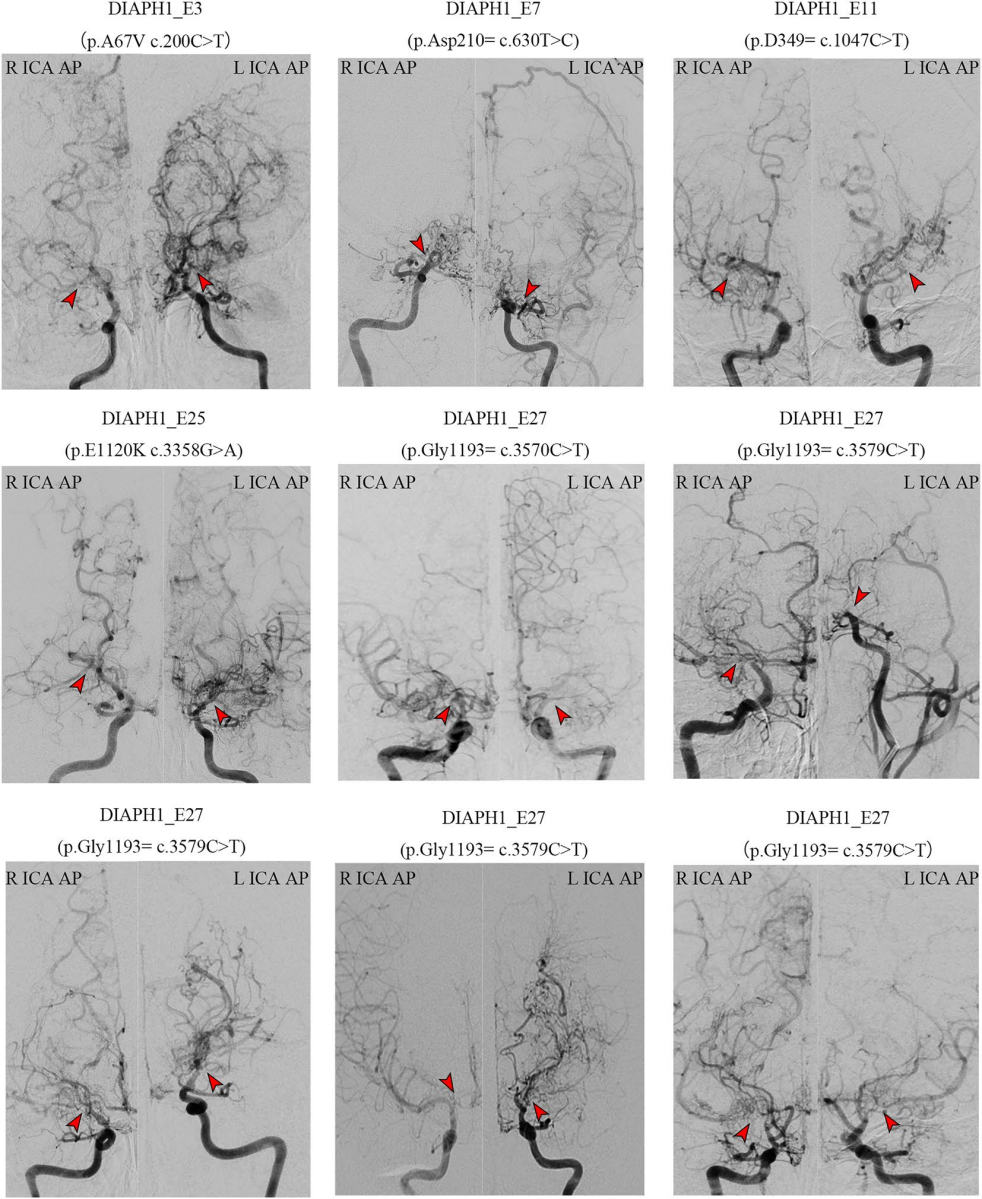
The anteroposterior (AP) view of internal carotid artery (ICA) angiography of 9 MMD patients with DIAPH1 mutation. What red arrows indicate is the typical MMD vascular manifestation.

**Figure 2:**
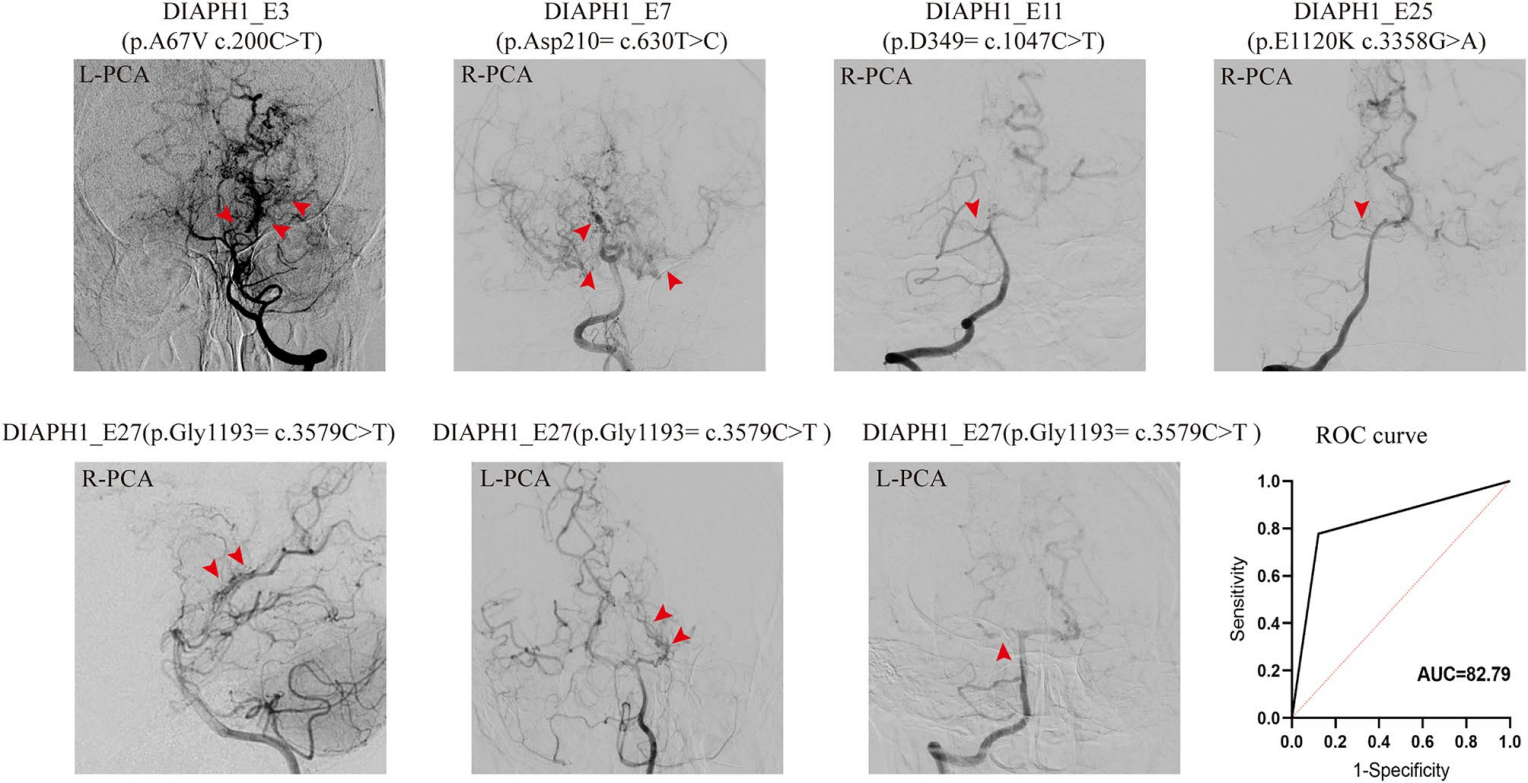
Six anteroposterior and one lateral (No. 6) vertebral arteriography showed typical PCA involvement in 7 patients in the group of DIAPH1mutation. The red arrow shows the involved posterior cerebral artery. Areas under the ROC curve of DIAPH1 mutation as a independent risk factor.

The DSA image of 9 MMD patients with DIAPH1 gene mutation is shown in Fig. 1, with each image is named by their exon fragments. Among them, 7 cases had typical PCA involvement (Fig. 2), while only 5 cases in the group without genetic mutation were involved, the constituent ratio of the former (77.8%) was significantly higher than that of the latter (12.2%) (*p* = 0.001), but there was no significant statistical difference in age, sex, initial symptoms, platelet count, BMI, Suzuki stage and so on (Table 2).

**Table 2:** Characteristics between different group.

Variables	Group 1, with DIAPH1 variant (n = 9)	Group2, without DIAPH1 variant (n = 41)	Statistics p values
Baseline data			
Sex (M : F)	6:3	19:22	0.462
Age, years, median (IQR)	35 (10–36)	23 (9–34)	0.245
History of risk factors, n (%)			
Hypertension	1 (11%)	3 (10%)	1.000
Diabetes	0	0	1.000
Coronary heart disease	0	0	1.000
Hyperlipidemia	0	0	1.000
Smoking history	1 (11%)	2 (4.9%)	1.000
Alcohol taking	0	0	1.000
Clinical manifestation, n (%)			0.291
TIA	3 (11%)	5 (11%)	
Infarction	3 (11%)	22 (11%)	
Hemorrhage	3 (11%)	14 (11%)	
Clinical features, mean (SD)			
BMI, kg/m^2^	23.25 ± 6.13	22.50 ± 5.27	0.709
PLT count, 10^9^/L	240.11 ± 51.84	263.66 ± 59.60	0.279
PCA involvement (+), n (%)	7 (77.8%)	5 (12.2%)	0.001
Leptomeningeal (−)^a^ (posterior to anterior), n (%)	4 (44.5%)	8 (19.5%)	0.248
Unilateral MMD, n (%)	0	2 (4.9%)	1.000
Suzuki stage			
Left			0.319
1	0	1 (2.6%)	
2	0	4 (10.3%)	
3	2 (22%)	10 (25.6%)	
4	3 (33%)	10 (30%)	
5	3 (33%)	9 (23.1%)	
6	1 (11%)	5 (12.8%)	
Right			0.853
1	0	1 (2.4%)	
2	1 (11%)	0	
3	2 (22%)	16 (39.0%)	
4	3 (33%)	13 (31.7%)	
5	3 (33%)	8 (19.6%)	
6	0	3 (7.3%)	

IQR, interquartile range; TIA, transient ischemia attack; BMI, Body Mass Index; PLT, platelet; PCA, posterior cerebral artery.

^a^Without compensation of leptomeningeal from posterior to anterior.

### Logistic regression analysis of variables associated with PCA involvement

Between groups with and without PCA involvement, there were significant statistical differences in DIAPH1 mutation and compensation of leptomeningeal from posterior to anterior. DIAPH1 mutation was more frequently observed in the PCA involvement than in the PCA non-involvement group (58.3% versus 5.3%; *p* = 0.001). Compensation of leptomeningeal from posterior to anterior was more frequently observed in the PCA non-involvement than in the PCA involvement group (58.3% versus 5.3%; *p* = 0.001). But there were no significant differences in sex, age, first symptom and clinical features. Multivariate logistic regression analysis of risk factors for PCA involvement showed that after adjusting for sex, age, platelet count, there is an association between DIAPH1 mutation and PCA involvement (OR 29.483; 95% CI 3.920–221.736; p = 0.001) (Table 3), areas under the ROC curve is 82.79 (Fig. 2).

**Table 3:** Logistic regression analysis of predictors for PCF.

Characteristics	PCA		p values		OR (95% CI)
	Involvement, n (%) (n = 12)	Non-involvement, n (%) (n = 38)	Univariate	Multivariate	
DIAPH1 variant	7 (58.3%)	2 (5.3%)	0.001	0.001	29.483 (3.920–221.736)
Baseline data					
Sex (M : F)	6:6	18:20	0.874	0.462	1.950 (0.329–11.553)
Age, years, median (IQR)	19.5 (12–37.25)	26.5 (9–34)	0.717	0.834	1.007 (0.942–1.077)
Clinical manifestation, n (%)					
TIA	2 (16.7%)	6 (15.8%)	0.936		
Infarction	6 (50%)	19 (50%)	0.997		
Hemorrhage	4 (33.4%)	13 (34.2%)	0.954		
Clinical features, mean (SD)					
BMI, kg/m^2^	23.3 ± 6.1	22.5 ± 5.2	0.630		
PLT count, 10^9^/L	243.6 ± 56.8	267.7 ± 62.0	0.236	0.480	1.006 (0.990–1.021)
Leptomeningeal (−)^a^ (posterior to anterior), n (%)	7 (58.3%)	5 (13.2%)	0.005		

IQR, interquartile range; BMI, Body Mass Index; PLT, platelet; PCA, posterior cerebral artery.

^a^Without compensation of leptomeningeal from posterior to posterior to anterior.

## Discussion

The main purpose of this study is to explore the genetic background of moyamoya disease in different ethnic groups. We have identified a different Asian Moyamoya disease and European DIAPH1 mutation with a site associated with posterior circulation involvement.

As reported by Kundishora et al.^9^, DIAPH1 is a novel moyamoya disease risk gene in non-East Asian patients with moyamoya disease. They conducted a genetic association study using whole-exome sequencing. We found two new mutations (p.A67V, c.200C>T in exon3; p.E1120K, c.3358G>A on exon 25) of the DIAPH1 gene in two Asian MMD. DIAPH1 is a formin protein encoding the RhoA GTPase mDia^10^, which can stimulates actin filament assembly at the barbed ends after being activated by GTP-bound RhoA^11^. DIAPH1 induces platelet formation in megakaryocytes by regulating actin and microtubule cytoskeleton^12^, which could causes macrothrombocytopenia and extends the spectrum of DIAPH1-related disease^13^. DIAPH1 also mediates vascular remodeling by integrating oxidative stress and signal transduction pathways in smooth muscle cells. In cardiovascular and cerebrovascular diseases, DIAPH1 gene mutations increase the genetic susceptibility to the risk of cerebral ischemic stroke^8^. Several studies have proposed the effect of DIAPH1 on angiogenesis from different perspectives^14^. Studies published in mice lacking DIAPH1 have shown that the mouse is protected from hyperdilation of the femoral intima/medium after endothelial stripping, and has reduced infarct size and loss of cardiac function after ligation of the coronary artery in the left anterior descending branch of the heart^15^. And the deletion of DIAPH1 can reduce infarct size after the occurrence of myocardial infarction^16^.

The Receptor for Advanced Glycation End Products (RAGE) is expressed by multiple cell types in the brain and spinal cord that are associated with the mechanism of neurovascular and neurodegenerative disorders, such as vascular cells (endothelial cells, smooth muscle cells and pericytes), neurons and glia (microglia and astrocytes)^17^. Current structural and functional evidence points to the interaction of the RAGE cytoplasmic domain with the forming protein DIAPH1 as a key cytoplasmic hub for RAGE ligand-mediated cell signaling activation. A SNP rs1035798 gene variant of RAGE was found to be significantly associated with the subtype of small vessel disease (SVD) (OR 1.56, 95% CI 1.16–2.09), adjusted p-value < 0.05), and the association was independent of hypertension, diabetes, and smoking^18^.

We found a very interesting phenomenon that the probability of PCA involvement in the DIAPH1 gene mutation group was significantly higher than that in the non-mutation group (77.8% versus 12.2%, p = 0.000). PCA involvement could affect the leptomeningeal collateral flow from the PCA to anterior circulation, which could influence clinical outcomes^19^. These patients often had more severe anterior circulation stenosis and more likely to have cerebral infarction, which often led to bad prognosis^20^. About 1/3 of MMD patients had PCA involvement^21^. Previous studies have proved that genetic factors can affect the clinical manifestations of moyamoya patients, for example, the pR4810K polymorphism of RNF213 gene is closely related to the risk of MMD. Patients with AA genotype have earlier onset age and PCA involvement than those with GG and GA genotypes^22,23^, and the first symptoms are more likely to be cerebral infarction^24^. DIAPH1 is a key effector of actin remodeling in vascular cells and platelets^13^, which mediates vascular remodeling by integrating oxidative stress and signal transduction pathways in smooth muscle cells^8^. Furthermore, it has been reported that to a lesser extent DIAPH1 silencing has disrupted stress fibers in endothelial cells. And miR-24 targets on DIAPH1 downstream of Rho signaling, therefore repressing the actin polymerization through Profilin^14^. Therefore, similar to the effect of RNF213 on angiogenesis, we speculate that MMD patients with DIAPH1 gene deficiency can produce vascular intima and media thinning induced by ischemia, so blood vessels are vulnerable to hemodynamic stress and secondary injury, which may promote the involvement of posterior cerebral artery.

In addition, there were limitations in this study. The relatively small sample size may deviate the interpretation of cerebrovascular changes, so it is necessary to improve the statistical efficiency to determine the accuracy of the results. In future studies, a larger, multi-ethnic sample size is needed to determine the significance of DIAPH1 mutations in MMD. Nevertheless, this study has certain clinical significance to clarify the cerebrovascular damage of MMD under different genetic backgrounds.

## Conclusion

In conclusion, it is premature to conclude an association between DIAPH1 gene and MMD, especially in Asian patients. However, we found a strong association between DIAPH1 gene mutation and PCA involvement.

## Methods

The data that support the findings of this study are available from the corresponding author on reasonable request.

### Study design and inclusion and exclusion criteria

The whole blood samples used in this study were obtained from patients admitted to Beijing Tiantan Hospital from October 2020 to August 2022. Patients were eligible if (1) they were diagnosed with unilateral or bilateral MMD by digital subtraction angiography (DSA), following the lastest Japanese guidelines^25^; (2) under the age of 50; (3) agree to participate in the study and provide a blood sample; (4) without serious underlying diseases that could affect the results of the study. Patients were not eligible if (1) they refused to participate in the study; (2) without DSA; (3) lack of laboratory data; (4) Moyamoya syndrome with thalassemia, Down syndrome and systemic immune system disease. The study was approved by the Institutional Review Board of Beijing Tiantan Hospital. This experiment was approved by the Ethics Committee of Beijing Tiantan Hospital (KY 2020-045-02) and all methods were performed in accordance with the relevant guidelines and regulations. Informed consents were obtained from patients or their representatives.

### Baseline data collection

Information on sex, age, first symptoms, ethnicity, place of birth, BMI, platelet count, DSA data were collected. The first symptoms were transient ischemic attack (TIA), cerebral infarction and cerebral hemorrhage. Besides, peripheral blood samples were collected after admission for all patients. Levels of routine and biochemical blood tests were measured using enzymatic methods.

### Classification and evaluation of angiographic variables

Suzuki stage, posterior cerebral artery (PCA) involvement, compensation of leptomeningeal from posterior to anterior were evaluated. If the occlusion or stenosis of P1–P3 segments of unilateral or bilateral PCA is confirmed by digital subtraction angiography, PCA involvement is considered^26^. Two experienced neurosurgeons (Y.Z. and R.W.) independently evaluated the angiographic findings.

### Variant detection of DIAPH1

Refer to Relax Gene Blood DNA system Instruction Book, genomic DNA from patients was extracted from peripheral blood samples using a Magnetic bead method genome extraction kit (NMG0121-100, CHINA). Design primers for DIAPH1 gene using Primer Premier 5 software, all primers are in supplementary materials 2. The primer sequence of DIAPH1 gene was amplified by polymerase chain reaction (PCR) amplification instrument, the PCR reaction conditions are as follows: pre-denaturation at 96 °C last for 5 min; denaturation at 96 °C for 20 s; annealing at 60 °C for 20 s; chain extension at 72 °C for 30 s, amplification for 35 cycles, and finally supplementary extension at 72 °C for 10 min. The PCR system is 25 μL. 3 μL PCR products were detected by 1.0% agarose gel electrophoresis, and then purified according to the standard operating procedure of magnetic bead purification. The purified product was sequenced by ABI3730xL sequencer. The data was processed using the phred\phrap software.

### Statistical analysis

Statistics analyses were performed using SPSS (version 26.0) and GraphPad Prism (version 9.4). Measurements that matched the normal distribution were presented as X ± SD, the independent sample t test was used for the comparison between groups. Measurements that did not matched the normal distribution were presented as median and interquartile range; the Mannwhitney U test was used for the comparison between groups. The counting data was expressed as frequency and percentage, and the comparison between groups was compared with χ^2^ test or Fisher accurate test. Multivariate logistic regression analysis was used to determine the independent risk factors of PCA involvement. Two-sided test p < 0.05 was statistically significant.

## Supplementary Information

Supplementary Information 1.

Supplementary Information 2.

## Data Availability

The data that support the findings of this study are available from the corresponding author on reasonable request.

## References

[1] Suzuki J, Takaku A (1969). Cerebrovascular, “moyamoya” disease. Disease showing abnormal net-like vessels in base of brain. Arch. Neurol..

[2] He S, Duan R, Liu Z, Ye X, Yuan L, Li T, Tan C, Shao J, Qin S, Wang R (2020). Characteristics of cognitive impairment in adult asymptomatic moyamoya disease. BMC Neurol..

[3] Hallemeier CL, Rich KM, Grubb RL, Chicoine MR, Moran CJ, Cross DT, Zipfel GJ, Dacey RG, Derdeyn CP (2006). Clinical features and outcome in North American adults with moyamoya phenomenon. Stroke.

[4] Ihara M, Yamamoto Y, Hattori Y, Liu W, Kobayashi H, Ishiyama H, Yoshimoto T, Miyawaki S, Clausen T, Bang OY (2022). Moyamoya disease: Diagnosis and interventions. Lancet Neurol..

[5] Guo DC, Papke CL, Tran-Fadulu V, Regalado ES, Avidan N, Johnson RJ, Kim DH, Pannu H, Willing MC, Sparks E (2009). Mutations in smooth muscle alpha-actin (ACTA2) cause coronary artery disease, stroke, and Moyamoya disease, along with thoracic aortic disease. Am. J. Hum. Genet..

[6] Roy V, Ross JP, Pepin R, Cortez Ghio S, Brodeur A, Touzel Deschenes L, Le-Bel G, Phillips DE, Milot G, Dion PA (2022). Moyamoya disease susceptibility gene RNF213 regulates endothelial barrier function. Stroke.

[7] Keylock A, Hong Y, Saunders D, Omoyinmi E, Mulhern C, Roebuck D, Brogan P, Ganesan V, Eleftheriou D (2018). Moyamoya-like cerebrovascular disease in a child with a novel mutation in myosin heavy chain 11. Neurology.

[8] Ren ZY, Chen XT, Tang WZ, Li J, Yang S, Chen YC, Zhao XH, Zong HH, Liu CL, Shen C (2020). Association of DIAPH1 gene polymorphisms with ischemic stroke. Aging-Us..

[9] Kundishora AJ, Peters ST, Pinard A, Duran D, Panchagnula S, Barak T, Miyagishima DF, Dong W, Smith H, Ocken J (2021). DIAPH1 variants in Non-East Asian patients with sporadic moyamoya disease. JAMA Neurol..

[10] Shimada A, Nyitrai M, Vetter IR, Kuhlmann D, Bugyi B, Narumiya S, Geeves MA, Wittinghofer A (2004). The core FH2 domain of diaphanous-related formins is an elongated actin binding protein that inhibits polymerization. Mol. Cell.

[11] Goode BL, Eck MJ (2007). Mechanism and function of formins in the control of actin assembly. Annu. Rev. Biochem..

[12] Pan JJ, Lordier L, Meyran D, Rameau P, Lecluse Y, Kitchen-Goosen S, Badirou I, Mokrani H, Narumiya S, Alberts AS (2014). The formin DIAPH1 (mDia1) regulates megakaryocyte proplatelet formation by remodeling the actin and microtubule cytoskeletons. Blood.

[13] Stritt S, Nurden P, Turro E, Greene D, Jansen SB, Westbury SK, Petersen R, Astle WJ, Marlin S, Bariana TK (2016). A gain-of-function variant in DIAPH1 causes dominant macrothrombocytopenia and hearing loss. Blood.

[14] Zhou Q, Anderson C, Zhang H, Li X, Inglis F, Jayagopal A, Wang S (2014). Repression of choroidal neovascularization through actin cytoskeleton pathways by microRNA-24. Mol. Ther..

[15] Toure F, Fritz G, Li Q, Rai V, Daffu G, Zou YS, Rosario R, Ramasamy R, Alberts AS, Yan SF (2012). Formin mDia1 mediates vascular remodeling via integration of oxidative and signal transduction pathways. Circ. Res..

[16] O'Shea KM, Ananthakrishnan R, Li Q, Quadri N, Thiagarajan D, Sreejit G, Wang LJ, Zirpoli H, Aranda JF, Alberts AS (2017). The formin, DIAPH1, is a key modulator of myocardial ischemia/reperfusion injury. EBioMedicine.

[17] MacLean M, Derk J, Ruiz HH, Juranek JK, Ramasamy R, Schmidt AM (2019). The receptor for advanced glycation end products (RAGE) and DIAPH1: Implications for vascular and neuroinflammatory dysfunction in disorders of the central nervous system. Neurochem. Int..

[18] Olsson S, Jood K (2013). Genetic variation in the receptor for advanced glycation end-products (RAGE) gene and ischaemic stroke. Eur. J. Neurol..

[19] Kim JM, Lee SH, Roh JK (2009). Changing ischaemic lesion patterns in adult moyamoya disease. J. Neurol. Neurosurg. Psychiatry.

[20] Huang AP, Liu HM, Lai DM, Yang CC, Tsai YH, Wang KC, Yang SH, Kuo MF, Tu YK (2009). Clinical significance of posterior circulation changes after revascularization in patients with moyamoya disease. Cerebrovascul. Dis. (Basel, Switzerl.).

[21] Hishikawa T, Tokunaga K, Sugiu K, Date I (2013). Assessment of the difference in posterior circulation involvement between pediatric and adult patients with moyamoya disease. J. Neurosurg..

[22] Miyatake S, Miyake N, Touho H, Nishimura-Tadaki A, Kondo Y, Okada I, Tsurusaki Y, Doi H, Sakai H, Saitsu H (2012). Homozygous c.14576G>A variant of RNF213 predicts early-onset and severe form of moyamoya disease. Neurology.

[23] Kamada F, Aoki Y, Narisawa A, Abe Y, Komatsuzaki S, Kikuchi A, Kanno J, Niihori T, Ono M, Ishii N (2011). A genome-wide association study identifies RNF213 as the first Moyamoya disease gene. J. Hum. Genet..

[24] Liu W, Morito D, Takashima S, Mineharu Y, Kobayashi H, Hitomi T, Hashikata H, Matsuura N, Yamazaki S, Toyoda A (2011). Identification of RNF213 as a susceptibility gene for moyamoya disease and its possible role in vascular development. PLoS ONE.

[25] Kuroda S, Fujimura M, Takahashi J, Kataoka H, Ogasawara K, Iwama T, Tominaga T, Miyamoto S (2022). Diagnostic criteria for moyamoya disease—2021 revised version. Neurol. Med. Chir. (Tokyo).

[26] Funaki T, Takahashi JC, Houkin K, Kuroda S, Takeuchi S, Fujimura M, Tomata Y, Miyamoto S (2018). Angiographic features of hemorrhagic moyamoya disease with high recurrence risk: A supplementary analysis of the Japan Adult Moyamoya Trial. J. Neurosurg..

